# Effect of moderate livestock grazing on soil and vegetation characteristics in zokor mounds of different ages

**DOI:** 10.1038/s41598-023-39530-7

**Published:** 2023-08-01

**Authors:** Qian Zhang, Yan Lu, Caijun Zhang, Baohui Yao, Junhu Su

**Affiliations:** 1 College of Grassland Science, Key Laboratory of Grassland Ecosystem (Ministry of Education), Gansu Agricultural University, Lanzhou, 730070 China; 2 Gansu Agricultural University-Massey University Research Centre for Grassland Biodiversity, Gansu Agricultural University, Lanzhou, 730070 China

**Keywords:** Grassland ecology, Restoration ecology

## Abstract

Mounds formed by plateau zokors (*Eospalax baileyi*) in alpine meadows are easily disturbed by livestock. We aimed to reveal the effect of moderate livestock grazing (from October 15 to March 15 of the following year) on plant and soil characteristics of zokor mounds. This study explored the effect of zokor mounds of different ages (2015–2018) on soil nutrient content, soil enzymatic activity, plant diversity, and aboveground biomass (AGB) at grazing and non-grazing sites. Compared with the non-grazing sites, soil organic carbon (SOC), total soil phosphorus, and ratio of SOC to total nitrogen were 16.6%–98.7% higher and soil urease activity was 8.4% and 9.6% higher in 1- and 3-year-old mounds, respectively, at the grazing sites. Grazing significantly increased the plant Pielou index, richness, and Shannon–Wiener diversity index of 4-year-old mounds by 20.7%–52.4%. Partial least squares path modeling showed that plant species diversity was the main factor affecting the plant AGB of mounds at the grazing sites, whereas soil enzyme activity was the primary factor at the non-grazing sites. We propose that moderate grazing increases soil nutrient content and the plant diversity in zokor mounds in alpine meadows, which should be considered in future grassland restoration.

## Introduction

Grasslands account for 40% of the world’s land area^1^. More than 90% of grasslands in China are thought to be degraded by overgrazing^2^. Similar problems are reported throughout Central Asia in the vast Eurasian grasslands^3^. In addition, underground rodents can cause the degradation of grasslands as they continuously excavate underground tunnels and deposit soil aboveground^4,5^. These activities can have both negative and positive effects on vegetation and soil properties, especially when coupled with livestock in the grassland^6^.

The plateau zokor, a small underground herbivorous rodent, feeds on the subsurface roots and stems of plants in the 0–20 cm soil layer and brings soil to the surface through digging activity, resulting in the formation of bare mounds in grasslands^5,7^. Bare mounds are different from the vegetation of the intact grassland. Because of the formation time of mounds, different ages of zokor mounds coexist in grasslands, which may increase plant diversity. First, if new plants colonize continuously, the plant diversity of zokor mounds increases^8^. Second, plant diversity is highest if the plant community contains many short-lived plants and long-lived species sensitive to disturbance^9^. Variation in plant composition and diversity directly alters soil organic carbon (SOC) content, other nutrient content, and soil enzyme activity^10^. As the age of zokor mounds increases, vegetation height decreases, the soil becomes more compact, and the soil water content and organic matter decomposition rate change^11^. These observations indicate that the role of zokor mounds should not be neglected in the study of fodder–livestock balance and sustainable grassland utilization.

Livestock grazing can affect the vegetation of zokor mounds. First, livestock grazing could reduce seed production due to consumption of palatable plants which they did not form seed^12^. Second, some species seeds (such as *Galium aparine*) can be dispersed in plant communities by livestock grazing, especially in grassland, and seedling establishment is a crucial stage at which the bare zokor mounds are initiated^13^. Grazing also reduced the spatial heterogeneity of soil seed banks compared with non-grazing sites^14^. Moreover, livestock grazing accelerates the decomposition of leaves and roots by trampling activity. Meanwhile, livestock dung and urine alter soil nutrient content and the activity of enzymes (such as sucrase, urease, and phosphatase)^15^, which can lead to a varied environment for hosting a diverse range of annual and perennial plants. Thus, livestock grazing alters the soil characteristics of zokor mounds.

Grassland management should evaluate the role and significance of mound-forming rodents in the ecosystem^11^. Optimizing the interaction between livestock grazing and mound-forming rodents is important for successfully managing grasslands. Recent studies have focused on two aspects. The first is the influence of livestock grazing on zokor density by changing soil and plant properties^16–18^. For example, Harris et al. (2015)^19^ found that zokor density is positively correlated with grazing intensity because the dominant plants of degraded grassland were preferred by plateau zokors, such as *Potentilla anserina*, *Geranium wilfordii*, and *Taraxacum mongolicum*^20^. Conversely, grazing destroys tunnels and the food resources of rodents and reduces their population in grasslands^11^. The second aspect is that zokor disturbance positively affects ecosystem function in non-grazing grasslands. For example, Su et al. (2020)^21^ found that zokor disturbance had a positive effect on soil microbial communities, and Zhang et al. (2003)^11^ reported that the plateau zokor, an important ecosystem engineer, enhanced grassland heterogeneity and the rate of infiltration of soil water, thereby reducing soil erosion.

The effective management of livestock and small rodents is important for preserving the grassland ecosystem^11,22^. Therefore, this study investigated the relationship between plateau zokor mounds and livestock grazing to promote the sustainable utilization of grasslands. We answer the following questions: (1) how do soil nutrient content, soil enzymatic activity, and plant characteristics change in zokor mounds of different ages at grazing and non-grazing sites, and (2) how does livestock grazing change the relationship between aboveground plant biomass (AGB) and plant species diversity, soil nutrient content, and enzymatic activity?

## Materials and methods

### Study area

**Figure 1:**
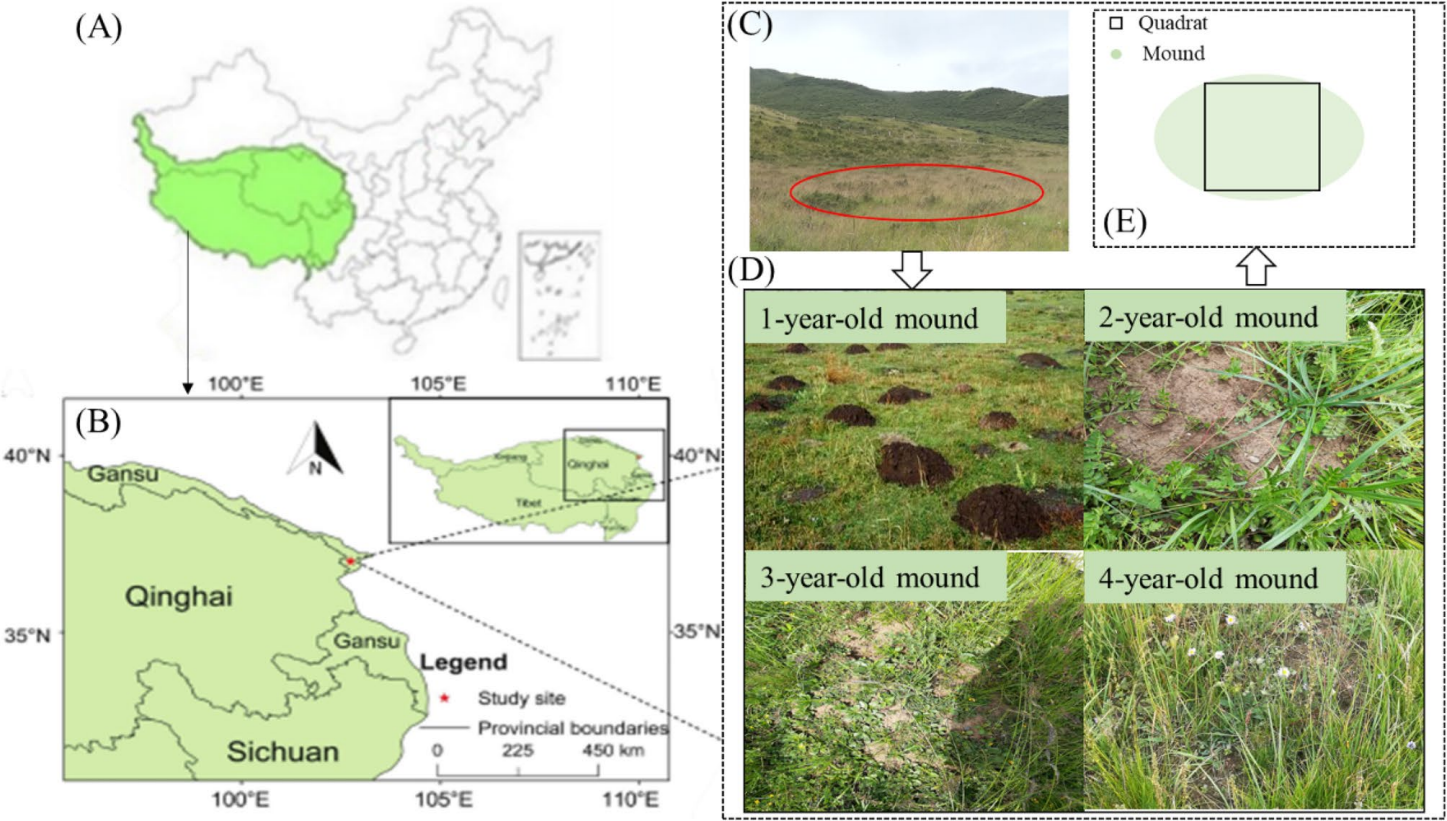
(**A**) Location of the Tibetan Plateau in China. (**B**) Location of the study site in the Tibetan Plateau. (**C**) The red solid line in the image shows the distribution of mound age at the site. (**D**) Image showing the mounds of different ages. (**E**) Schematic quadrat of the zokor mounds.

The location of the study area was in Zhuaxixiulong Town, Tianzhu County, Gansu Province, China, with geographical coordinates of 37°10′–37°13′ N, 102°45′–102°48′ E (Fig. 1A,B). The average altitude is 2700–3100 m and the mean annual temperature is − 0.1 °C. The mean annual precipitation is 416 mm, with more than 60% occurring between June and August. There are approximately 120 days of plant growth from May to September. A continental semi-arid climate characterizes the study area, and the soil type is classified as alpine chernozem under the soil classification system of the United States Department of Agriculture^23^.

### Experimental design

Based on the field investigations and after consultation with local farmers, two nearby non-grazing and grazing sites were selected in which the plateau zokor was the only underground rodent^11^. The altitude, geographic coordinates, and area of two sites are presented in Table 1. One site was established as non-grazing for more than 10 years (the goal of grazing removal), and the other site was established as grazing with a moderate stocking rate of 4–5 sheep per ha (from 15 October to 15 March of the following year)^24^ (Table 1). We selected population sizes of plateau zokor that were the same at non-grazing and grazing sites, as described by Zhang et al. (2003)^11^. Basic information on the vegetation and soil condition is presented in Table 2. All zokor mounds within each site were marked annually from 2015 to 2018 and classified into four different ages based on our previous study^21^. The 1-, 2-, 3-, and 4-year-old mounds were constructed in 2018, 2017, 2016, and 2015, respectively (Fig. 1C,D). Moreover, detailed information on the vegetation structure and soil characteristics in the zokor mounds is presented in Table 2^25^.

**Table 1:** The altitude, geographic coordinates, areas, grazing period, and grazing rate of the study sites.

Sites	Altitude (m)	Geographic coordinates	Areas (km^2^)	History (year)	Grazing period	Stocking rate (sheep per ha)	Reference
Grazing	2938	102°45′36′′ E 37°12' N	0.044	> 5	Grazing from 15 October to 15 March of the next year	4.0–5.0	Ortega et al., 1997
Non-grazing	2910	102°46′48′′ E 37°12′ N	0.054	> 10	Completely excluded from grazing	0	Ortega et al., 1997

**Table 2:** Vegetation and soil conditions before the experiment started at the grazing and non-grazing sites. TN and TP represent soil total nitrogen and soil total phosphorus, respectively.

Sites	Soil nutrient content			Vegetation conditions		
	Soil layers (cm)	TN (g kg^−1^)	TP (g kg^− 1^)	Total coverage (%)	Average height (cm)	Important value
Grazing	0–10	5.03 ± 0.69	0.50 ± 0.17	80	14.33 ± 1.63	1.31 ± 0.13
	10–20	3.63 ± 0.06	0.54 ± 0.03			
	20–30	3.50 ± 1.07	0.41 ± 0.10			
Non-grazing	0–10	4.87 ± 0.19	0.52 ± 0.07	89	16.54 ± 2.32	1.40 ± 1.01
	10–20	3.43 ± 0.16	0.50 ± 0.12			
	20–30	3.80 ± 1.27	0.40 ± 0.15			

### Sampling and measurements

#### Survey of community characteristics and soil sampling

To measure the number of plant species and plant coverage in the mounds, we set up six portable quadrats (50 × 50 cm) in mounds of each age at the grazing and non-grazing sites in August 2019 (Fig. 1E). Plant AGB was clipped at ground level in each quadrat, and dry matter was weighed after drying at 80 °C^26^. Because the plateau zokor feeds on the subsurface roots and stems of plants in the 0–20 cm soil layer and brings soil to the surface through digging activity^7^, soil samples were therefore collected from the 0–20 cm soil layer. Six soil cores were collected for mounds of each age using a soil auger (5 cm in diameter).

#### Soil physical, chemical properties, and soil enzyme activity analysis

All soil samples were passed through a 0.25 mm sieve and stored at 4 °C for the measurement of nutrients and enzyme activity. The dichromate oxidation method was used to determine soil organic carbon (SOC), the Kjeldahl method was used to determine soil total nitrogen (TN), and the HClO_4_–H_2_SO_4_ method was used to determine soil total phosphorus (TP)^27^. The urease activity was determined using the indophenol blue colorimetric method, the alkaline phosphatase activity was determined using the benzene disodium phosphate method, and sucrase activity was determined using the 3,5-dinitrosalicylic acid colorimetric method^28^.

### Ethical approval

This study obtained appropriate permissions from the natural resource bureau of Tianzhu Tibetan Autonomous County to enter the study area and for the collection of any plant and soil material; thus, we complied fully with the relevant institutional, national, and international guidelines and legislation. The vegetation survey was recorded and collected in the field, but no recorded species were Species at Risk of Extinction. Therefore, we fully complied also with the IUCN Policy Statement on Research Involving Species at Risk of Extinction and the Convention on the Trade in Endangered Species of Wild Fauna and Flora.

### Statistical analysis

The plant species richness, Pielou, Simpson, and Shannon–Wiener indices were calculated from the following formulas^29^. The study also calculated the differences between the grazing and the non-grazing grassland on mound of each age by the relative response indices^30,31^ (RRIs) of plant diversity.

1 $$Richness=S$$

2 $$Pielou=\frac{-\sum ({P}_{i}\times \mathrm{ln}{P}_{i})}{\mathrm{ln}S}$$

3 $$Simpson={\sum_{i=1}^{S}P}_{i}^{2}$$

4 $$Shannon-Wiener=-\sum ({P}_{i}\times \mathrm{ln}{P}_{i})$$

5 $${\text{RRI }} = \, \left( {{\text{X}}_{{\text{g}}} - {\text{ X}}_{{\text{n}}} } \right)/\left( {{\text{X}}_{{\text{g}}} + {\text{ X}}_{{\text{n}}} } \right)$$

*S* is the sum of species in each quadrat, *P*_*i*_ = *N*_*i*_*/N*, *N* = ***∑N***_*i*_*,* and *N*_*i*_ is the individuals per species; X_g_ is the value of a dependent variable (diversity diversity) in the grazing site; and X_n_ is the value of the same dependent variable in the non-grazing site paired with the adjacent grazing site. The values of RRI range between − 1 and + 1. The closer |RRI| is to 1, the higher the difference between the grazing and non-grazing grasslands.

Origin 2021 software was used to analyze the effect of plant AGB on soil nutrient content (TN, TP, and SOC) and plant species diversity (Simpson and Shannon–Wiener indices) at grazing and non-grazing sites through a general linear model.

The soil nutrients, soil enzymatic activity (urease, alkaline phosphatase, and sucrase), and plant characteristics (Simpson index, Shannon–Wiener index, Pielou index, plant species richness, plant AGB, plant total coverage, annual species proportion, and perennial species ratio) were presented as the mean ± standard error, and the differences between the zokor mounds of different ages were analyzed by one-way analysis of variance for the grazing and non-grazing sites. An independent sample *t*-test was conducted to analyze the differences in soil nutrient content, soil enzymatic activity, and plant characteristics between grazing and non-grazing sites for each mound age. These analyses were performed using the SPSS 2021 software, and all data conformed to the normal distribution and homogeneity of variance.

This study performed partial least squares path modeling (PLS-PM) using the PLS-PM package in R software (Version 4.0.2) to find the direct and indirect driving factors affecting the plant AGB of zokor mounds. The model comprises two sub-models: (1) the measurement model in which the observable variables were related to their latent variables; for example, plant total coverage, proportion of annual species, and proportion of perennial species were defined as observable variables, and their corresponding latent variable was mound age; and (2) the path model in which some latent variables were associated with other latent variables.

## Results

### Changes in soil properties

#### Changes in soil nutrient content

**Figure 2:**
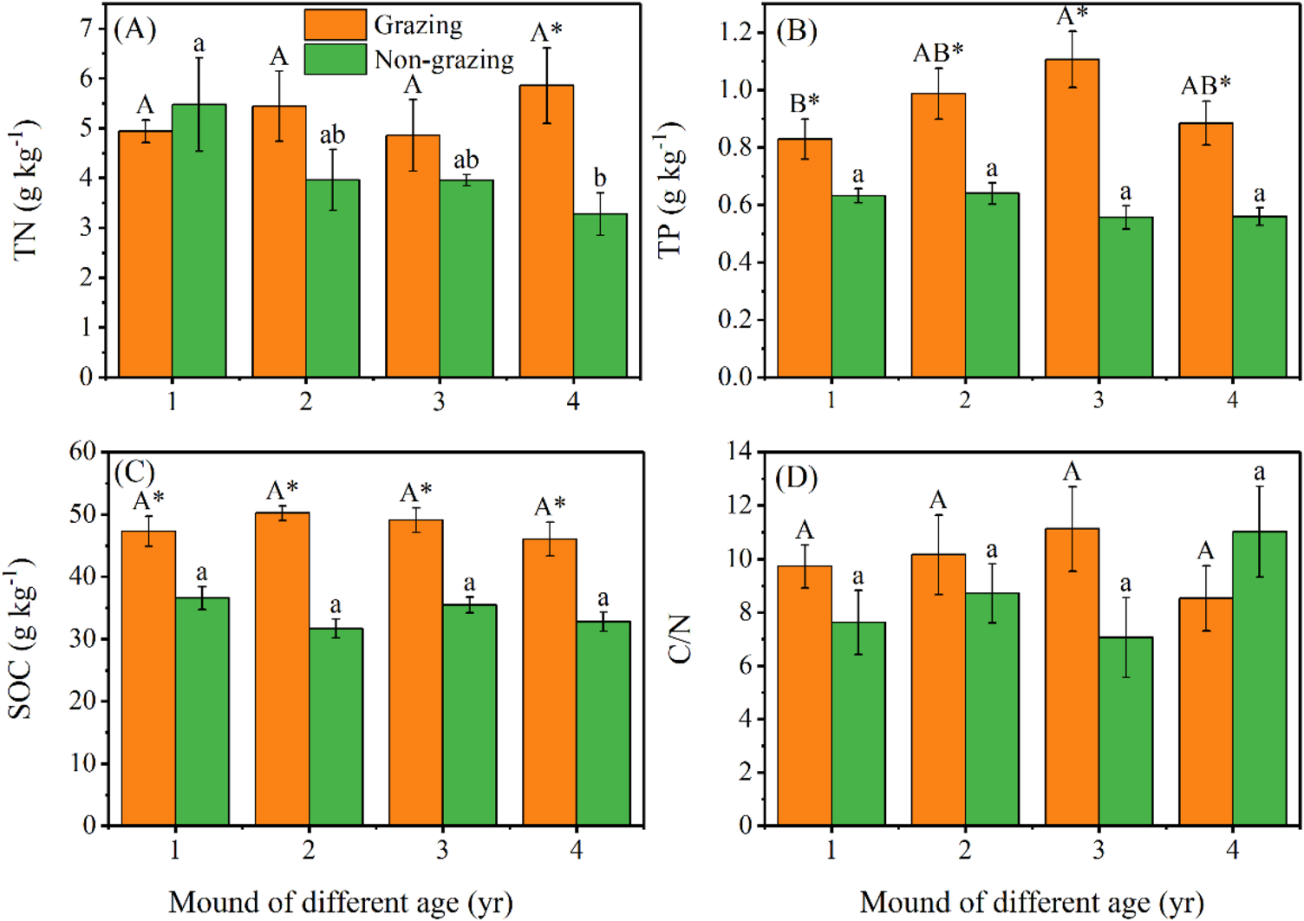
Variation in TN (**A**), TP (**B**), SOC (**C**), and C/N (**D**) in mounds of different ages. TN, TP, SOC, and C/N represent soil total nitrogen, soil total phosphorus, soil organic carbon, and ratio of soil total nitrogen to organic carbon, respectively. Uppercase (grazing) and lowercase (non-grazing) letters represent significant differences by mound age at *P* < 0.05; * represents a significant difference between grazing and non-grazing sites at *P* < 0.05 for the same mound age. Bars and errors represent the mean and standard error.

At the non-grazing sites, the TN content decreased by 40.1% as mound age increased and TP content decreased by 11.6% (Fig. 2A,B); the SOC to TN ratio (C:N) increased by 44.7% from 1- to 4-year-old mounds; and the SOC content decreased by 10.4% as mound age increased (Fig. 2D). At the grazing sites, the TN content increased by 18.5%; TP and SOC increased by 33.5% and 2.6% from 1- to 4-year-old mounds, respectively; and C:N increased by 14.1% from 1- to 3-year-old mounds (Fig. 2A,B,D).

Compared with the non-grazing sites, the grazing sites had significantly higher TN content in 4-year-old mounds (78.6%) (Fig. 2A). Moreover, grazing significantly increased TP content in 2- (54.2%), 3- (98.7%), and 4-year-old (58.2%) mounds. In addition, C:N at the grazing sites was 27.6%, 16.6%, and 57.7% higher in 1-, 2-, and 3-year-old mounds, respectively. The content of SOC at the grazing sites was 29.3%, 58.6%, 38.4%, and 40.5% higher in 1-, 2-, 3-, and 4-year-old mounds, respectively (Fig. 2B,C,D).

#### Changes in soil enzyme activity

**Figure 3:**
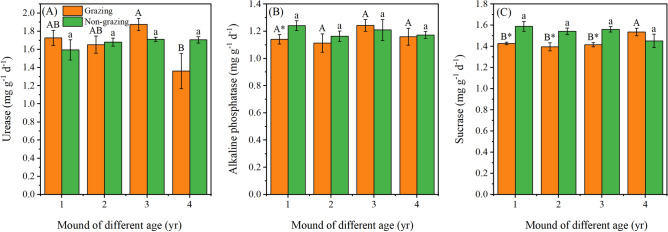
Variation in urease (**A**), alkaline phosphatase (**B**), and sucrase (**C**) activity in mounds of different ages. Uppercase (grazing) and lowercase (non-grazing) letters represent significant differences by mound age at *P* < 0.05; * represents significant differences between grazing and non-grazing sites at *P* < 0.05 for the same mound age. Bars and errors represent the mean and standard error.

At the non-grazing sites, from 1- to 4-year-old mounds, soil urease activity was increased by 6.9%, sucrase activity decreased by 8.6%, and alkaline phosphatase activity fluctuated slightly, ranging from 1.25 to 1.15 mg g^−1^ d^−1^. At the grazing sites, urease activity decreased by 21.2% from 1- to 4-year-old mounds, and alkaline phosphatase activity fluctuated slightly, ranging from 1.10 to 1.25 mg g^−1^ d^−1^, and sucrase activity increased by 7.6% from 1- to 4-year-old mounds (*P* < 0.05) (Fig. 3A,C).

Compared with the non-grazing sites, urease activity was increased at the grazing sites by 8.4% and 9.6% in 1- and 3-year-old mounds, respectively. However, sucrase activity decreased by 10.1% (*P* < 0.05), 9.4% (*P* < 0.05), and 9.2% (*P* < 0.01) in 1- to 3-year-old mounds, respectively. Moreover, soil alkaline phosphatase activity in 1-year-old mounds was lower at the grazing sites than at the non-grazing sites (*P* < 0.05) (Fig. 3B).

### Changes in vegetation characteristics

#### Plant families

**Figure 4:**
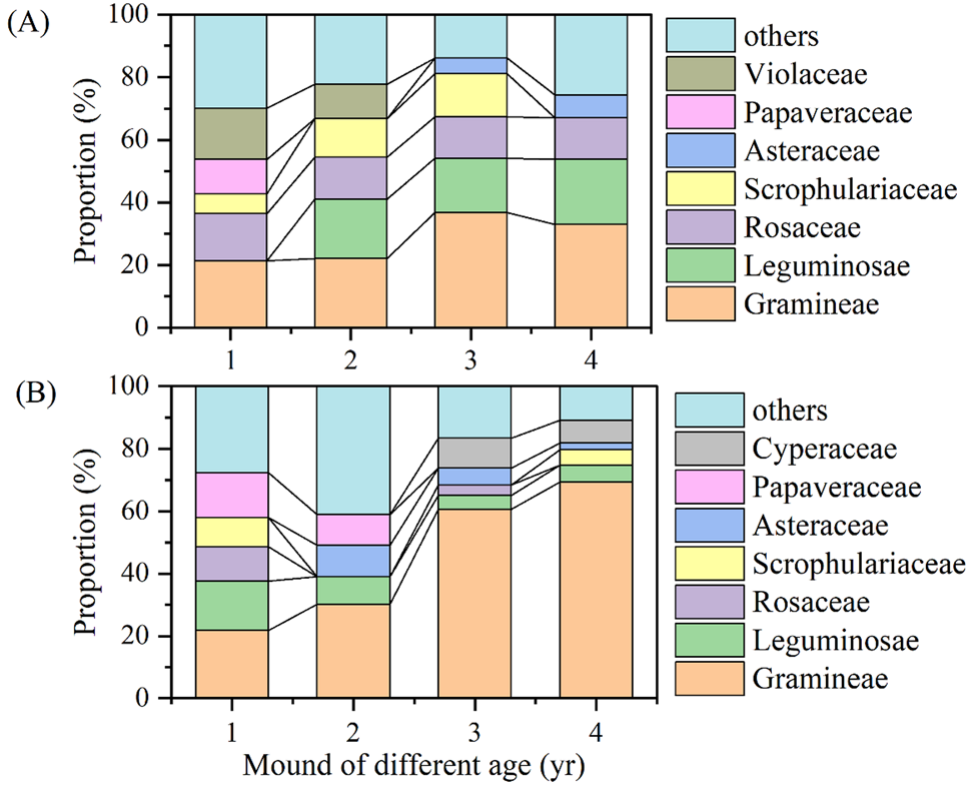
Variation in plant families at the grazing (**A**) and non-grazing (**B**) sites.

**Figure 5:**
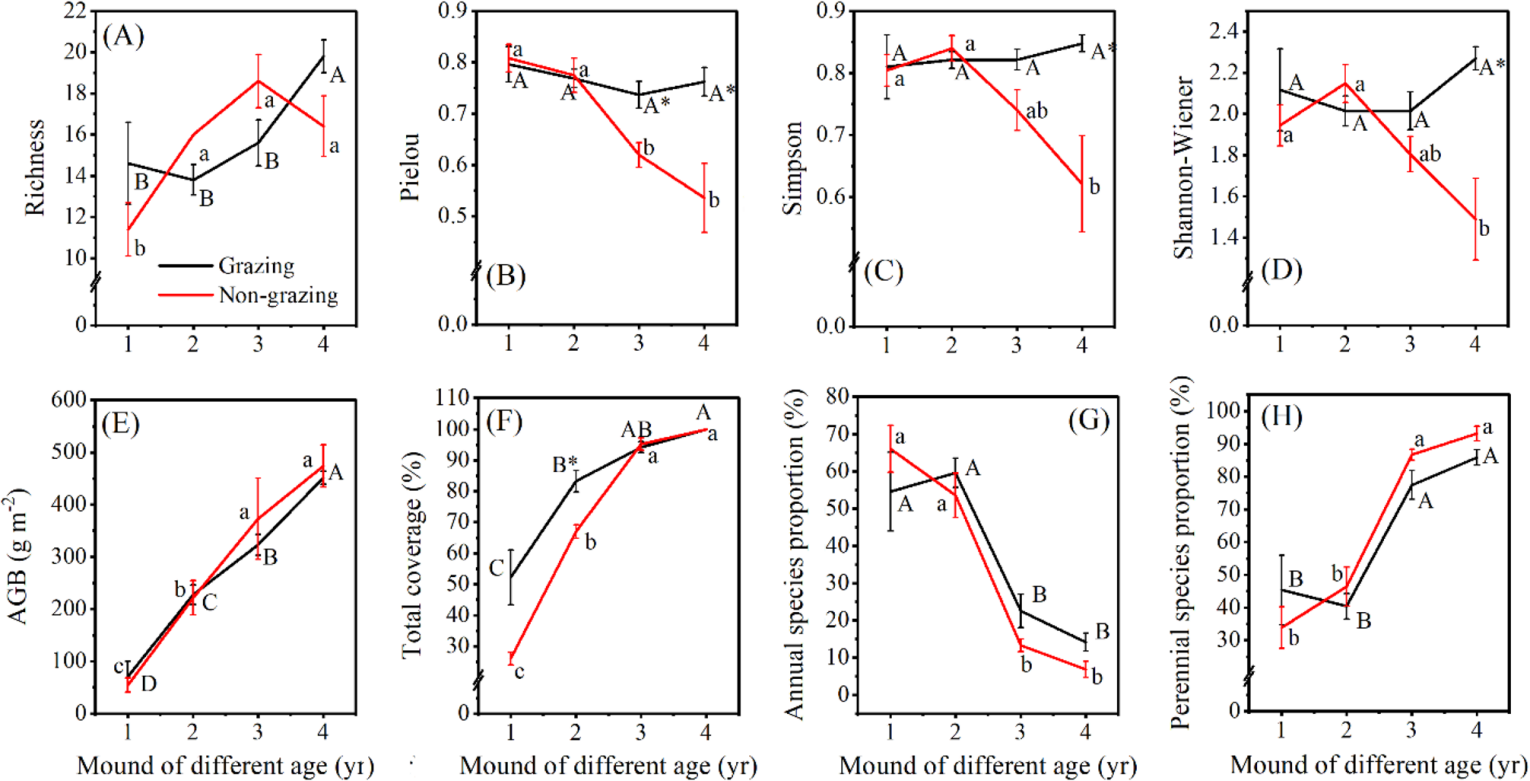
Variation in plant richness (**A**), Pielou index (**B**), Simpson index (**C**), Shannon–Wiener index (**D**), AGB (**E**), total coverage (**F**), annual species proportion (**G**), and perennial species proportion (**H**) in mounds of different ages. AGB represents the aboveground biomass. Uppercase (grazing) and lowercase (non-grazing) letters represent significant differences by mound age at *P* < 0.05; * represents significant differences at *P* < 0.05 between grazing and non-grazing sites for the same mound age.

Variations in vegetation communities at the family level at the grazing (Fig. 4A) and non-grazing sites (Fig. 4B) were investigated. As mound age increased, the proportion of Gramineae increased at the grazing and non-grazing sites. Gramineae (28.3%) and Leguminosae (14.3%) were the dominant families at the grazing sites; Gramineae (45.4%) was dominant at the non-grazing sites. Compared with the non-grazing sites, the proportion of gramineous species was 37.7% lower at the grazing sites, and that of leguminous species was 64.8% higher. As mound age increased, the proportion of perennials increased (Fig. 5F,H), whereas that of annual plant species decreased (Fig. 5G). Plant species of 1- and 2-year-old zokor mounds were different such as *Viola striatella* and *Hypecoum erectum*, respectively, at grazing and non-grazing sites; but the main plant species are gramineae (*Elymus nutans, Poa annua*, and *Thalictrum aquilegiifolium*) in 3- and 4-year-old mounds (Table S1).

#### Plant species diversity

At the non-grazing sites, as mound age increased, the plant Pielou, Simpson, and Shannon–Wiener indices decreased by 33.7% (*P* < 0.05) (Fig. 5B), 22.8% (Fig. 5C), and 23.4%, respectively (Fig. 5D). At the grazing sites, the plant Simpson index increased from 1- to 4-year-old mound ranging from 0.81 to 0.85. In addition, plant richness was higher in 4-year-old mounds than in 1-, 2-, and 3-year-old mounds (*P* < 0.05). The plant Shannon–Wiener index increased from 2.12 in 1-year-old mounds to 2.27 in 4-year-old mounds (Fig. 5A).

Compared with the non-grazing sites, the grazing sites had a higher plant Pielou index for 3-year-old mounds of 18.9% (*P* < 0.01) and 4-year-old mounds of 42.2% (*P* < 0.05). Grazing increased plant richness, the Shannon–Wiener index, and the Simpson index of 4-year-old mounds by 20.7%, 52.4%, and 36.4%, respectively. As mound age increased, plant AGB and total coverage increased (Fig. 5E). From the RRIs, the RRIs calculated for richness, Pielou index, Simpson index, and Shannon–Wiener index were the highest for 4-year-old mounds followed by 3-year-old mounds (Table 3).

**Table 3:** The relative response indices (RRIs) of each mound age in grazing and non-grazing grasslands.

Zokor mound ages	Richness	Pielou index	Simpson index	Shannon–Wiener index
1-year-old	0.1106	− 0.0084	− 0.0001	0.0349
2-year-old	− 0.0765	− 0.0025	− 0.0110	− 0.0314
3-year-old	− 0.0877	0.0865	0.0535	0.0552
4-year-old	0.1001	0.18690	0.1675	0.2208

### Relationships between plant AGB, soil nutrients, and plant species diversity

**Figure 6:**
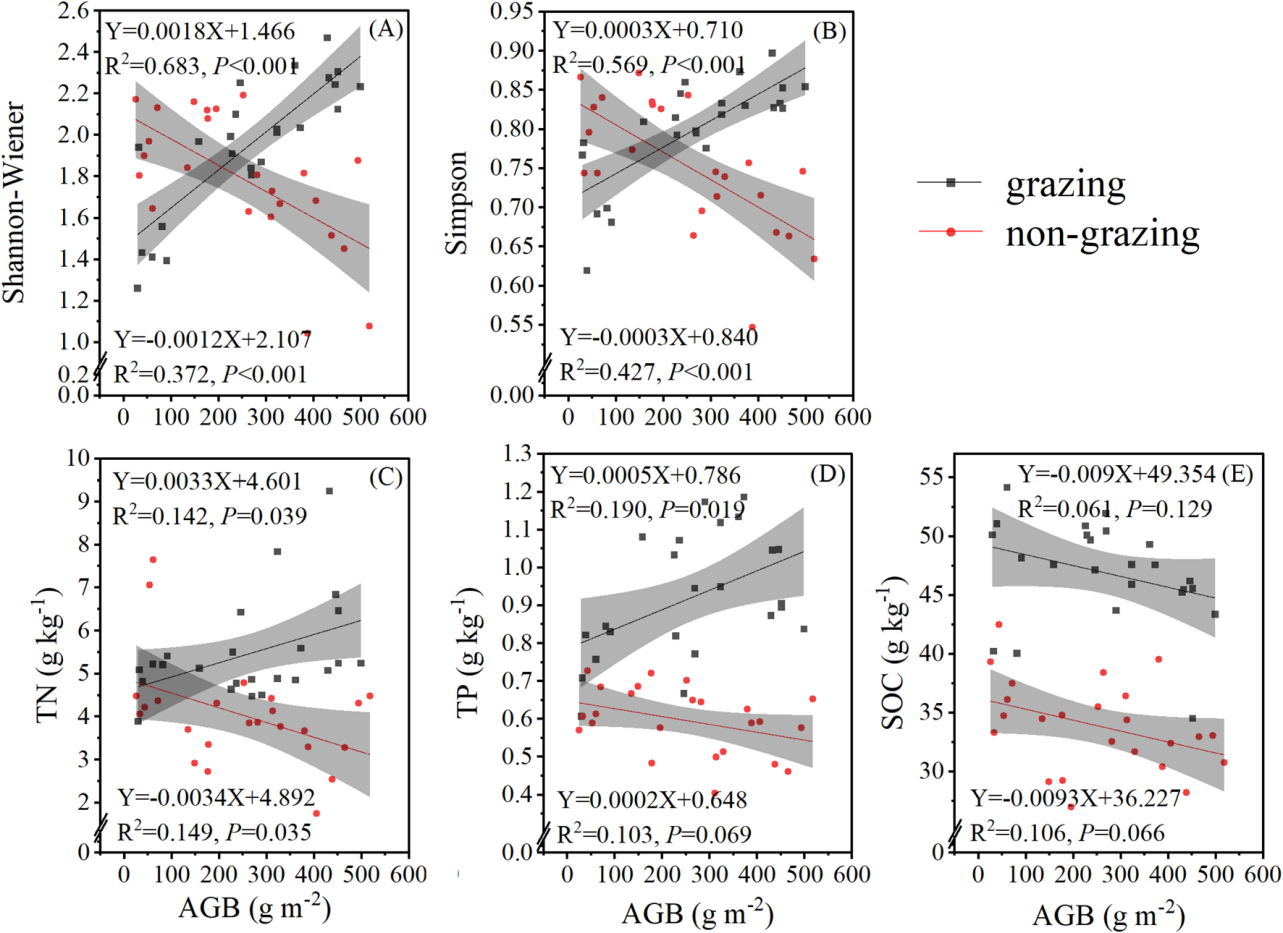
Relationship between the AGB and Shannon–Wiener (**A**) and Simpson (**B**) indices, TN (**C**), TP (**D**), and SOC (**E**) in mounds of different ages at the grazing and non-grazing sites. AGB, TN, TP, and SOC represent plant aboveground biomass, soil total nitrogen, soil total phosphorus, and soil organic carbon, respectively.

For the grazing sites, linear regression could simulate the relationship between plant AGB and the Simpson index (*P* < 0.001) and the Shannon–Wiener index (*P* < 0.001). The plant Simpson index (Fig. 6A), Shannon–Wiener index (Fig. 6B) were positively correlated with the plant AGB, and the TN (Fig. 6C) and TP (Fig. 6D) were positively correlated with plant AGB (*P* < 0.05).

For the non-grazing sites, linear regression could simulate the relationship between plant AGB and the Simpson index (*P* < 0.001) and the Shannon–Wiener index (*P* < 0.001). The plant Simpson index (Fig. 6A) and Shannon–Wiener index (Fig. 6B) were negatively correlated with plant AGB, and the TN (Fig. 6C), TP (Fig. 6D), and SOC (Fig. 6E) were negatively correlated with plant AGB (*P* < 0.05, *P* > 0.05, and *P* > 0.05, respectively).

### Effects of mound age on AGB at grazing and non-grazing sites

**Figure 7:**
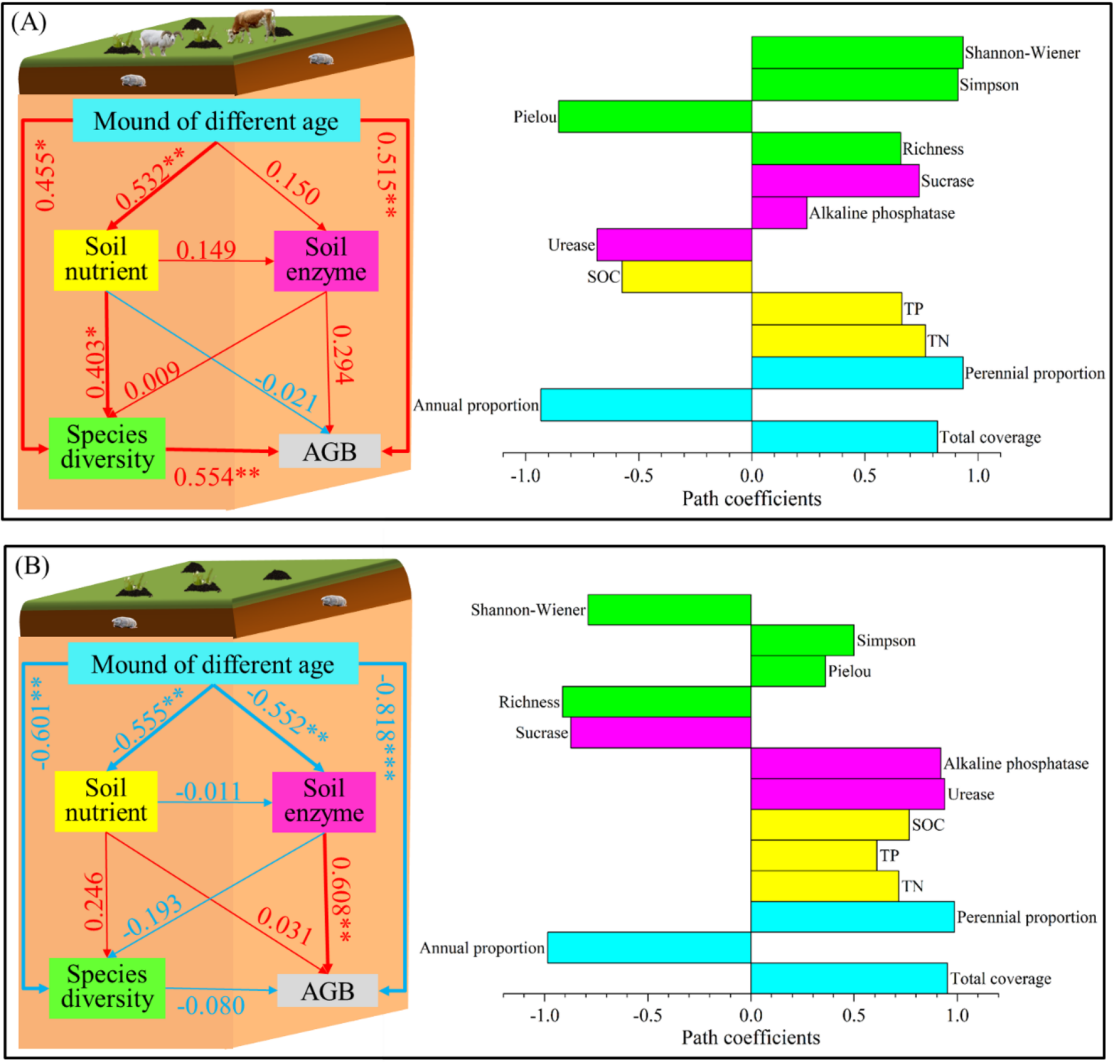
Partial least squares path modeling (PLS-PM) was used to analyze the direct and indirect effects of mound age on plant AGB at the grazing (**A**) and non-grazing (**B**) sites. AGB, TN, TP, and SOC represent plant aboveground biomass, soil total nitrogen, soil total phosphorus, and soil organic carbon, respectively. The red and blue lines indicate positive and negative influences, respectively. The width of the line represents the significance level. Numbers represent standardized path coefficients; *, **, and *** represent *P* < 0.05, *P* < 0.01, and *P* < 0.001, respectively.

The effects of mound age on plant AGB through soil nutrients, soil enzyme activity, and plant species diversity were detected using partial least squares path modeling (PLS-PM). At the grazing sites (Fig. 7A), mound age had significant positive effects on plant AGB through plant species diversity and directly affected the plant AGB with a path coefficient of 0.515 (*P* < 0.01). At the non-grazing sites (Fig. 7B), mound age had significant adverse effects on plant AGB through effects on soil enzyme activity and directly affected the plant AGB with a path coefficient of − 0.818 (*P* < 0.001). In addition, mound age had significant adverse effects on soil nutrients, soil enzymes, and plant species diversity, with path coefficients of − 0.555, − 0.552, and − 0.601, respectively (*P* < 0.01).

## Discussion

### Effect of livestock grazing on soil nutrients and enzyme activity in mounds of different ages

Livestock grazing can have direct effects on soil nutrient content owing to trampling activity and excrement. Our study showed that moderate livestock grazing significantly increased SOC content compared with non-grazing; this was consistent with the results of Sun et al. (2021)^32^ that indicated moderate grazing could improve soil nutrient content in healthy alpine grasslands. Grazing can lead to biomass transfer from aboveground to belowground and thus increase the organic matter returned to the soil^32,33^. However, a long-term (> 30 years) grazing experiment found that grazing activity (light grazing, medium grazing, and high grazing) decreased the SOC, TN, and TP content in alpine meadows^34^. The main reason is that selective consumption by long-term livestock increases the proportion of forbs with lower decomposability^35,36^. These changes likely reduce soil organic matter input and further decrease soil nutrient availability.

These results indicate that the trampling activity of livestock grazing accelerates the soil nutrient cycling process and the decomposition rate of organic matter^37^. The soil nutrient content is closely related to litter input and roots in the soil, and the increased litter biomass may be the primary source of soil nutrients^38^. Our results also showed that the TN and TP content increased with the increase in plant AGB at the livestock grazing sites. Livestock grazing directly affects the amount of litter and root inputs into the soil. First, litter is fragmented from trampling by livestock, which accelerates the rate of litter decomposition in the soil^39^. Second, trampling activity brings deep soil roots to the surface and increases the input of organic material in the topsoil, ultimately increasing the nutrient content of the topsoil^40^, and livestock waste (manure and urine) also increases the nutrient content of the soil.

The soil C:N ratio can be used to estimate the decomposition and mineralization of organic matter^41^. Livestock grazing increased the soil C:N ratio in 1-, 2-, and 3-year-old mounds, which was different from the results of Pang et al. (2021)^42^, where plateau pika disturbance decreased the C:N ratio of bare soil. This variation could be attributed to differences in burrowing animal species. For example, some studies have indicated that the soil C:N ratio in mounds is slightly different because of the various soil carbon types and different body shapes of the small rodents that disturb the soil, for example, European rabbits (*Oryctolagus cuniculus*)^43^, pocket gophers (*Geomyidae* sp.)^44^, and plain vizcachas (*Lagostomus maximus*)^45^.

Concurrently, our study observed that the grazing sites had significantly lower soil sucrase activity in the 1-, 2-, and 3-year-old mounds compared with the non-grazing sites, which indicated a decline in the mineralization capacity of organic matter with animal urine input in the soil^46,47^. Furthermore, grazing decreased the proportion of gramineous species, leading to lower soil alkaline phosphatase activity. For example, in a previous study, it was shown that gramineous species cover was positively related to phosphatase activity^48^, indicating that the reduction in gramineous species at grazing sites may reduce hydrolyzed organic phosphorus to cope with conditions of phosphorus stress. However, soil urease activity in 3-year-old mounds was higher at the grazing sites than at the non-grazing sites because the animal urine input in soil could increase soil urease activity^49,50^.

### Effect of livestock grazing on plant diversity in mounds of different ages

Livestock grazing might change the plant species diversity in mounds. In this study, the plant Pielou, Shannon–Wiener, and Simpson indices of 3- and 4-year-old mounds were higher at the grazing sites. The intermediate disturbance hypothesis proposes that species diversity should be highest at intermediate grazing intensities and then decline at low or high levels of disturbance^51^. As grazing selectively removes palatable species^52^, it alters the plant community composition and may reduce competition, which promotes colonization by new species^53^. In addition, grazing facilitates seed dispersal and is conducive to growth and germination, which increases species coexistence^54^. For example, Zhang et al. (2009)^55^ reported that colonization by pioneer species and soil heterogeneity increase plant species richness in bare mounds. However, our study showed the plant AGB of mounds was not significantly different between the grazing and non-grazing sites because of the complementary biomass of different plant functional groups. Our results also confirmed that livestock grazing decreased the proportion of gramineous plants and increased the proportion of leguminous species. These results differ from the findings of a previous study that reported a reduction in forbs owing to their high palatability in desert grasslands. Selective feeding by sheep exhibits significant seasonal differences and is affected by grazing time and temperature^56^. The selective feeding of the same grass is different in different grassland types. For example, sheep prefer to eat *Aster pekinensis* when the plant community contains *Aster pekinensis* and *Leymus chinensis*; sheep do not like to eat *Aster pekinensis* when the plant community is rich in *Aster pekinensis* and *Lathyrus quinquenerviu*^57^.

Meanwhile, at the non-grazing sites, the plant Pielou, Simpson, and Shannon–Wiener indices decreased with mound age, which might be because the mound primarily contained Gramineae and the other families were less well-represented in the older ages^7,58^. Hence, plant species diversity decreased with mound age in the absence of livestock grazing in the grassland, which was similar to the study of Stephan et al. (2017)^59^ that found plant species were lost due to long-term enclosure. Conversely, grazing increased the plant species diversity of mounds, and a previous study found that grazing decreased plant species biodiversity in intact grasslands owing to livestock feed^34^. Thus, the combination of zokor mounds and grazing can maintain grassland plant diversity.

### Effect of livestock grazing on the relationship among soil nutrients, soil enzyme activities, plant species diversity, and plant AGB

As the plant AGB increased, the soil TN and TP content increased at the grazing sites. Livestock trampling accelerates the decomposition rate of plant litter^24,60^. Therefore, when the plant AGB increases, livestock trampling is conducive to increasing the soil TN, and the decomposition of livestock urine in the soil increases the soil TP^61^. At the non-grazing sites, as AGB increases, plant litter accumulates, but it decomposes slowly, leading to a decrease in the TN and TP^38^. This study shows that as the AGB increases, the Simpson and Shannon–Wiener indices increases at the grazing sites. Because moderate livestock trampling can inhibit the rapid growth of dominant species and provide opportunities for the growth of annual short-lived species, the diversity of grassland plant species increases^62^. In addition, each plant population in alpine meadows has its niche; however, only species with strong competitive abilities can coexist in a plant community^63^. This can limit the number of species, thereby contributing to the positive relationship between plant AGB and species diversity^64^. At the non-grazing sites, the plant Simpson and Shannon–Wiener indices were negatively correlated with the plant AGB. Some studies have also confirmed that disturbance by some small rodents, such as plateau zokors^7^, pocket gophers^65^, and Mongolian pikas^22^, is negatively correlated with plant species richness, although this differs from the effects of prairie dogs^66^ and mole rats^67^ on plant diversity. The differences in results were mainly caused by the plant species composition of grassland communities, the feeding habits of rodents, and the digging methods^68^.

This study shows that grazing can alter the relationship between the plant AGB and species diversity, soil nutrient content, and enzymatic activity. At the grazing sites, the mound age affected the plant AGB through plant species diversity. In contrast, at the non-grazing sites, mound age affected the plant AGB through soil enzyme activity. There are two primary reasons for this result. First, livestock foraging and trampling can decrease biomass through plant consumption. Plant consumption can stimulate the compensatory growth of the consumed plants^69^ or eliminate their apical dominance, resulting in the emergence of several new plants in a community^70,71^, thus contributing to an increase in plant species diversity and AGB. Second, plateau zokors are an underground rodent. Disturbance activities occur underground and directly affect soil properties, such as excavating plant roots, turning over soil, and bringing deep soil to the surface^72^. Zokor disturbance also changes the density and composition of soil seed banks as the disturbances bring seeds from the lower soil layer into the upper layer and provide suitable conditions for germination, especially in some sensitive habitats, such as alpine grasslands^73^. In brief, disturbance by plateau zokor affects plant AGB through the alteration of soil characteristics and soil seed banks, such as soil enzyme activity, at the non-grazing sites.

Previous knowledge indicates that the coexistence of livestock, such as cattle and sheep, with zokor is not beneficial for the development of healthy grasslands. Local governments have proposed that plateau zokor play a key role in grassland degradation^74^. However, a previous study found that when grazing intensity is low or moderate, high densities of zokor never occurs^11^. We explored the relationship between small burrowing mammals and moderate livestock grazing by examining the ecological role of mound-forming mammals in grasslands. To enhance soil nutrient content and plant species diversity, grazing intensity, modes, and time should be adjusted to achieve sustainable and stable grassland utilization.

## Conclusion

We investigated the effect of moderate grazing on soil nutrients, plant species diversity, and plant AGB in mounds of different ages. We found that moderate grazing significantly increased the SOC and TP content of the mounds. The higher SOC and TP of the mounds in grazed grasslands is a potential mechanism that determines which vegetation recovers naturally. The plant Pielou, Simpson, and Shannon–Wiener indices of 3- and 4-year-old mounds for the grazing sites were higher than those for the non-grazing sites, and moderate grazing increased plant species diversity, which is beneficial for restoring mound vegetation. However, soil sucrase activity was significantly lower in grazing sites than in non-grazing sites. Overall, this study found that plant species diversity is the key factor affecting the mound plant AGB in grazed grasslands, whereas soil enzyme activity is the key factor affecting non-grazed grasslands.

## Supplementary Information

Supplementary Table S1.

## Data Availability

The data that support the findings of this study are available from the corresponding author upon reasonable request.
