# Supplementary material for: Effect of moderate livestock grazing on soil and vegetation characteristics in zokor mounds of different ages
Source: Sci Rep. 2023 Aug 1;13:12459. doi: 10.1038/s41598-023-39530-7 (PMC10393963; doi:10.1038/s41598-023-39530-7)
Supplement: Supplementary file 1 — Supplementary Table S1. [file 41598_2023_39530_MOESM1_ESM.docx]

**Effect of moderate livestock grazing on** **soil and vegetation characteristics in zokor mounds of different ages**

Qian Zhang^a,b^, Yan Lu^a,b^, Caijun Zhang^a,b^, Baohui Yao^a,b^, Junhu Su^a,b^**^*^**

*^a^ College of Grassland Science, Key Laboratory of Grassland Ecosystem (Ministry of Education),* *Gansu Agricultural University, Lanzhou 730070, China.*

*^b^ Gansu Agricultural University-Massey University Research Centre for Grassland Biodiversity, Gansu Agricultural University, Lanzhou 730070, China.*

***Correspondence Author**: Prof. Junhu Su

Tel: +86-0931-7631213

E-mail: [sujh@gsau.edu.cn](mailto:sujh@gsau.edu.cn)

**Table S1** Dominant species from mounds of different ages in the grazing and non-grazing sites

| Zokor mound ages | Grazing | Non-grazing |
| --- | --- | --- |
| 1-year-old | *Elsholtzia ciliata*  *Viola striatella*  *Potentilla bifurca* | *E. ciliata*  *Hypecoum erectum*  *Bistorta vivipara* |
| 2-year-old | *Laneea tibetiea*  *Kochia scoparia*  *Chenopodium glaucum* | *Pocockia ruthenia*  *H. erectum*  *Aster tataricus* |
| 3-year-old | *Elymus nutans*  *Potentilla anserina*  *Poa annua* | *E. nutans*  *P. anserina*  *Thalictrum aquilegiifolium* |
| 4-year-old | *P. annua*  *T. aquilegiifolium*  *E. nutans* | *P. annua*  *T. aquilegiifolium*  *E. nutans* |
